# Surgical and Transcatheter Tricuspid Valve Interventions: An Electrophysiology-Focused Review

**DOI:** 10.3390/jcdd13040172

**Published:** 2026-04-19

**Authors:** Kevin S. Tang, Mark W. Abdelnour, Robert M. Tungate, Christina Mansour, Fabio Sagebin, Antonio H. Frangieh, David M. Donaldson

**Affiliations:** 1 Mary & Steve Wen Cardiovascular Division, University of California Irvine, Irvine, CA 92697, USA; abdelnou@hs.uci.edu (M.W.A.); afrangie@hs.uci.edu (A.H.F.);; 2 Division of Cardiology, University of California San Francisco, San Francisco, CA 94143, USA; 3 Division of Cardiology, Department of Medicine, Columbia University, New York, NY 10032, USA; 4 Division of Cardiothoracic Surgery, Department of Surgery, University of California Irvine, Orange, CA 92868, USA

**Keywords:** transcatheter tricuspid valve intervention, atrioventricular conduction system, conduction disturbances, lead management, tricuspid regurgitation

## Abstract

Increasing recognition of the clinical impact of isolated tricuspid regurgitation has led to rapid expansion of surgical and transcatheter tricuspid valve interventions. Given the close anatomic relationship between the tricuspid valve and the atrioventricular conduction system, both surgical and transcatheter approaches carry a significant risk of new conduction disturbances and permanent pacemaker implantation. A three-dimensional understanding of the atrioventricular conduction axis is essential to anticipate and mitigate these complications. This review provides a comprehensive overview of conduction system anatomy and physiology in the context of tricuspid valve interventions, highlighting the mechanisms underlying procedure-related conduction abnormalities. We also discuss contemporary management strategies, including approaches to pre-existing transvalvular leads, valve-sparing pacing alternatives, and the evolving role of electrophysiologists within the multidisciplinary heart team.

## 1. Introduction

Historically known as the “forgotten valve,” the tricuspid valve (TV) was often ascribed an inferior, secondary role in diseases of the left heart and pulmonary vasculature [1]. This paradigm has led to delayed referrals and poor outcomes with isolated surgical repair [2]. Recognition of the poor prognosis of untreated tricuspid regurgitation (TR) has accelerated the development of transcatheter tricuspid valve interventions (TTVI) [3,4]. Emerging clinical trial data support TTVI as a transformative therapy demonstrating significant improvements in functional capacity, quality of life, and reductions in heart failure-related hospitalizations [1,2].

As the landscape of TV therapy continues to evolve, increasing utilization of these interventions has brought renewed attention to procedure-related complications. One of the major risks for both surgical and TTVI modalities is the development of new conduction abnormalities requiring permanent pacemaker (PPM) insertion [5,6], along with the complex management of pre-existing transvalvular cardiac implantable electronic devices (CIEDs) leads [7]. In TRISCEND II, around 38.2% of patients treated with Evoque had a CIED lead at baseline [5]. In this context, the role of electrophysiologists in the management of postoperative conduction-related adverse events is becoming increasingly important in ensuring favorable patient outcomes. Accordingly, interdisciplinary heart teams must maintain a comprehensive understanding of TV anatomy and its adjacent conduction pathways to anticipate, prevent, and effectively address these adverse events. This review provides a comprehensive overview of cardiac conduction anatomy, explores the incidence and management of conduction disturbances following TV interventions, and discusses strategies for managing pre-existing transvalvular CIED leads.

## 2. Anatomy

The cardiac conduction system is vulnerable to iatrogenic injury during surgical and transcatheter interventions for structural heart diseases. A detailed understanding of the atrioventricular (AV) conduction axis and its relationship to surrounding cardiac structures, specifically TV, is therefore essential to optimize procedural safety and clinical outcomes.

### 2.1. Tricuspid Valve

**Figure 1:**
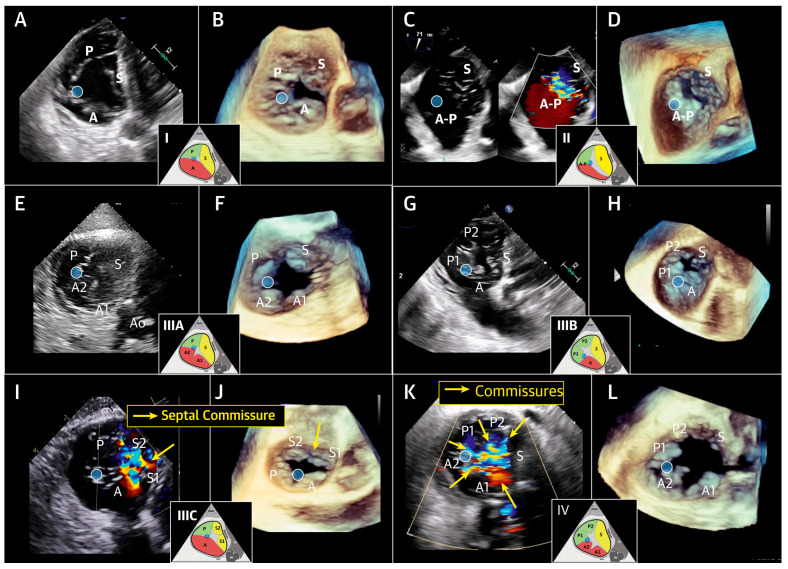
Transesophageal Imaging Examples of Tricuspid Valve Nomenclature Classification Scheme. In the examples shown in this Figure, locating the anterior papillary muscle (blue circle) defines the commissure between the anterior and posterior leaflets. (**A**,**C**,**E**,**G**,**I**,**K**) Transgastric 2-dimensional imaging plane; (**B**,**D**,**F**,**H**,**J**,**L**) 3-dimensional en face midesophageal view. (**A**,**B**) Type **I**, 3-leaflet configuration; (**C**,**D**) type **II**, 2-leaflet configurations; (**E**,**F**) type **IIIA**, quadricuspid valve with 2 anterior leaflet; (**G**,**H**) type **IIIB**, quadricuspid valve with 2 posterior leaflets; (**I**,**J**) **IIIC**, quadricuspid valve with 2 septal leaflets; (**K**,**L**) type **IV**, 5-leaflet configuration. A = anterior leaflet; P = posterior leaflet, S = septal leaflet. Reprinted/adapted with permission from Ref [8]. © 2021, Elsevier Inc. This is an Open Access article under the CC BY-NC-ND 4.0 license.

The TV apparatus is a dynamic and anatomically complex structure [5]. Classically, the TV consists of three leaflets: a small septal leaflet arising from the interventricular septum with multiple chordal attachments, a large and highly mobile anterior leaflet, and a posterior leaflet. However, contemporary anatomic studies demonstrate substantial variability, with four functional leaflets present in approximately 39% of cases, commonly comprising 2 posterior leaflets. Accordingly, a classification system based on leaflet number has been proposed: type I (three leaflets), type II (two leaflets), type III (four leaflets, with anterior, posterior, or septal duplication), and type IV (more than 4 leaflets) [5] (Figure 1).

The tricuspid annulus is nonplanar and elliptically shaped, with its posteroseptal segment positioned most ventricular and the anteroseptal segment most atrial. Two to three papillary muscles are typically present, with the anterior papillary muscle supporting the anterior and posterior leaflets and the posterior papillary muscle supporting the posterior and septal leaflets [5]. Several critical structures lie in close proximity to the TV, including the coronary sinus near the posteroseptal commissure, the right coronary artery within the AV groove, and the noncoronary sinus of Valsalva adjacent to the anteroseptal commissure [5]. Importantly, the AV node and right bundle of His course near the septal leaflet and anteroseptal commissure, underscoring the vulnerability of the conduction system during tricuspid valve intervention.

### 2.2. Hierarchical Phase-Contrast Tomography and the Atrioventricular Conduction Axis

**Figure 2:**
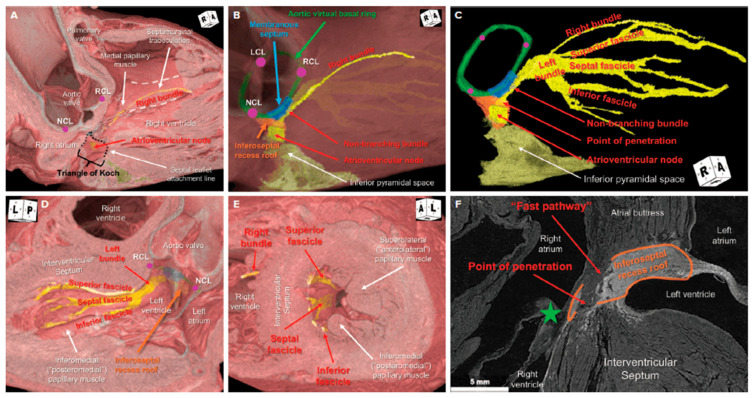
Three-dimensional anatomy of the atrioventricular conduction system revealed by hierarchical phase-contrast tomography (HiP-CT). (**A**–**C**) Right anterior oblique views. (**D**) Left three-chamber view. (**E**) Left anterior oblique projection demonstrating the segmented atrioventricular conduction axis. The atrioventricular node is positioned near the apex of the triangle of Koch, at the apex of the inferior pyramidal space. The conduction axis penetrates the rightward aspect of the roof of the inferoseptal recess (**C**), adjacent to the commissure between the anterosuperior and septal leaflets of the tricuspid valve (green star in (**F**)). Due to the obliquity of the section plane in (**F**), the cranial portion of the axis remains in myocardial continuity with the atrial septal buttress, consistent with the distal atrioventricular node. Only after complete insulation from the atrial myocardium does the axis form the non-branching bundle of His. The non-branching bundle courses along the inferior margin of the membranous septum, with the bundle branches arising anteriorly. The left bundle branch and its superior fascicle run adjacent to the nadir of the right coronary leaflet (RCL). The septal fascicle descends along the mid-septum, while the superior and inferior fascicles extend toward their respective papillary muscle insertions. The right bundle branch courses beneath the medial papillary muscle, continues along the septomarginal trabeculation, and traverses the moderator band to the anterior papillary muscle. LCL = left coronary leaflet; NCL = non-coronary leaflet; RCL = right coronary leaflet. Reprinted/adapted from Ref. [12]. © 2025, Radcliffe Medical Media. Licensed under the Creative Commons Attribution–Noncommercial 4.0 International (CC BY-NC 4.0) license.

The AV node was first reported by a Japanese pathologist, Dr. Sunao Tawara, in 1906 [9]. His work described the anatomical and functional features of the nodal tissue as part of the “impulse conduction axis of the heart”, where the atria and His-Purkinje system are connected [10]. Although early histologic observations were groundbreaking, their 2-dimensional (2D) nature limited correlation with the 3-dimensional (3D) anatomy of the heart. More recently, studies have leveraged the unprecedented spatial resolution of hierarchical phase-contrast tomography (HiP-CT) to enhance our understanding of the AV conduction axis [11,12]. HiP-CT enables 3D imaging at micron-level spatial resolution, allowing detailed visualization of conduction system anatomy. Figure 2 illustrates the 3D anatomy of the AV conduction system of an 80-year-old man, as revealed by a HiP-CT dataset [12]. Data derived from HiP-CT have made it possible to map the conduction axis onto conventional clinical CT datasets. Notably, the strong concordance between modern segmentation of the conduction axis and Tawara’s original anatomic descriptions validates this approach [12]. Although this work is currently limited to research applications, continued analysis of additional datasets may enable these methods to inform clinical practice, ultimately helping clinicians better identify and avoid the conduction system during clinical interventions.

### 2.3. Atrioventricular Conduction Axis

The 3D concept of a “Pyramid of Koch” (PoK) reveals the complex spatial relationships underlying the traditional triangle of Koch, providing a more accurate understanding of the location of the proximal AV components of the conduction axis [11]. This 3D perspective is crucial for clinicians seeking to accurately predict the location of the AV node to mitigate periprocedural complications.

**Figure 3:**
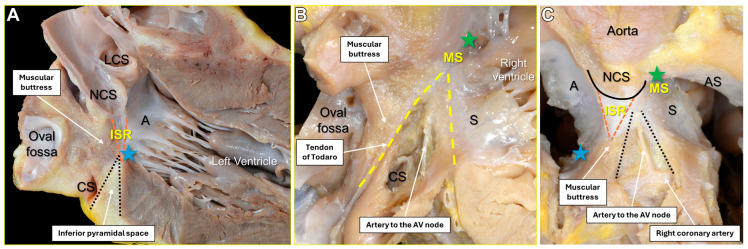
Dissections of a normal human heart demonstrating the three-dimensional anatomy underlying the pyramid of Koch. (**A**) Long-axis section of the left ventricle and aortic root illustrating the relationship between the inferior pyramidal space (black dashed lines), inferoseptal recess (orange hashed lines), and the inferomedial commissure of the mitral valve (blue star). (**B**) Rightward aspect of the atrial and ventricular septum with the triangle of Koch (yellow dashed lines); the floor of the triangle has been removed to expose the underlying inferior pyramidal space and the atrioventricular (AV) nodal artery. (**C**) Short-axis view of the mitral and tricuspid valves with dissection into the inferior pyramidal space, demonstrating its relationship to the inferoseptal recess, membranous septum (MS), inferomedial commissure of the mitral valve, and the commissure between the septal and anterosuperior leaflets of the tricuspid valve (green star). A = anterior mitral leaflet; AS = anterosuperior tricuspid leaflet; CS = coronary sinus; LCS = left coronary sinus; NCS = noncoronary sinus; S = septal tricuspid leaflet. Reprinted/adapted from Ref. [11]. © 2024, Elsevier Inc. Licensed under the Creative Commons Attribution–Noncommercial–NoDerivatives 4.0 International (CC BY-NC-ND 4.0) license.

The compact AV node resides at the base of the right atrium at the apex of the inferior pyramidal space, a fibro-adipose region corresponding to the 3D PoK. Rather than representing a planar structure, the AV node occupies a deep position adjacent to the hinge of the septal leaflet of the TV, which forms the apical border of the pyramid, and to the AV component of the membranous septum, where the lateral walls of the pyramid converge and contribute to the central fibrous body [11]. It is also closely related to the right fibrous trigone, with the right coronary sinus of the aortic root situated superiorly. This pyramidal structure is bounded inferiorly by the coronary sinus and the atrial vestibule between the anterior margin of the coronary sinus and the hinge of the septal tricuspid leaflet, forming a distinct paraseptal isthmus [11]. Figure 3 illustrates a dissection of a normal human heart anatomy showing the PoK.

From the compact node, located at the apex of the PoK, the AV conduction axis penetrates the central fibrous body through the roof of the inferoseptal recess. During this transition, it becomes electrically insulated from the surrounding atrial myocardium and continues as the non-branching His bundle [11]. This penetrating segment courses along the inferior margin of the membranous septum before reaching the crest of the muscular interventricular septum, where it bifurcates into the bundle branches.

### 2.4. Bundle Branches

The His bundle, first reported in 1893 by Wilhelm His Jr., is a single strand of specialized myocardium that provides electrical connection between the atria and ventricles [13]. It is anatomically divided into three parts: penetrating bundle of His, which penetrates the fibrous membranous septum close to the mitral ring and runs anteriorly and inferiorly; nonbranching part, which runs a variable course on the crest of the muscular interventricular septum; and branching part, which divides into the right and left bundle branches near the aortic annulus. The penetrating bundle represents the transition point from the AV node to the non-branching bundle, serving as the point of insulation of the axis before splitting into the bundle branches.

At the crest of the muscular septum, the non-branching bundle divides into the left and right bundle branches along the inferior margin of the membranous septum. The left bundle branch (LBB) typically originates anteriorly, adjacent to the nadir of the right coronary leaflet of the aortic valve and may lie within 3 mm of this landmark. It often courses subendocardial for 10–15 mm before dividing into superior, septal, and inferior fascicles, which, respectively, extend toward the superolateral papillary muscle, descend along the mid-septum, and course toward the inferomedial papillary muscle [11]. Although the branching morphology is highly variable, these spatial relationships remain consistent and are critical for understanding conduction disturbances during aortic and septal interventions.

The right bundle branch (RBB) arises as a slender cord at the inferior margin of the membranous septum and courses toward the medial papillary muscle before traveling within the septomarginal trabeculation and across the moderator band to the anterior papillary muscle [11]. Its superficial and anterior trajectory renders it particularly susceptible to injury during TV interventions. A 3D understanding of these conduction pathways, together with knowledge of device anchoring strategies, is essential to minimize clinically significant conduction disturbances [13].

## 3. Tricuspid Valve Surgical Repair and Replacement

### 3.1. Indications and Guidelines

**Figure 4:**
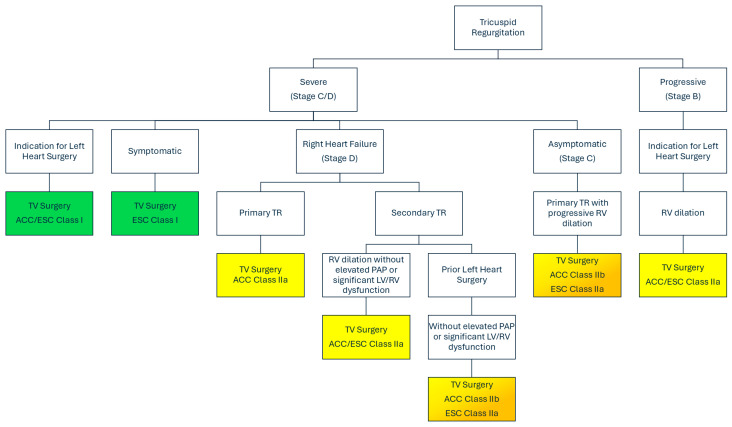
Society Guideline Indications for Tricuspid Valve Surgery in the setting of Tricuspid Regurgitation. ACC = American College of Cardiology; ESC = European Society of Cardiology; LV = left ventricle; PAP = Pulmonary arterial pressure; RV = right ventricle; TR = Tricuspid regurgitation; TV = Tricuspid valve.

Expert consensus from the 2025 ESC/EACTS and 2020 ACC/AHA guidelines for the management of patients with valvular heart disease maintains a Class I recommendation for TV surgery in patients with severe TR (Stages C or D) who have concomitant indication for left-sided valvular surgery [14,15]. Patients with severe TR are further recommended for isolated TV surgery with a range of Class I, IIa, and IIb depending on various factors, including TR etiology, symptom burden, and presence of right ventricle (RV) dilatation and elevated pulmonary arterial pressures, among others (Figure 4). TV surgery options include valve repair with or without annuloplasty or TV replacement with a bioprosthetic or mechanical valve [6,16]. Valve repair with annuloplasty is preferred over replacement whenever technically feasible, particularly in low-risk patients with favorable anatomy [17]. However, TV replacement may be necessary in cases of advanced disease with severe annular dilatation and leaflet tethering [7]. The long-term survival and clinical outcomes are similar between bioprosthetic and mechanical valve implants [18], with bioprosthetic options generally preferred due to a decreased risk of thromboembolic events [19]. Mechanical valves may still be considered in younger patients in light of the potential for long-term structural valve degeneration with bioprosthetic hardware [19]; choice of prosthesis should thus be guided by patient demographics and clinical presentation [20].

### 3.2. Indications for Postoperative Permanent Pacemaker Implantation

Postoperative conduction disorders are a major complication of TV surgery, although some data suggest that rates of new PPM implantation following TV surgery have decreased over the decades with improvement in materials and techniques [6]. A recent analysis of over 13,000 Medicare beneficiaries from 2013 to 2020 who underwent TV surgery found an overall incidence of 18.9% [21]. TV replacement carried a significantly high risk of postoperative PPM implantation when compared to TV repair (RR 3.20, *p* < 0.0001) because the vast majority of repairs involve incomplete annuloplasty rings, which spare the area over the AV node; other independent risk factors included female sex and preoperative second-degree AV block, RBB block, or bifascicular block [21]. Other studies have also suggested a higher risk of PPM implantation with concomitant left-heart valvular surgery and surgeries performed at a low-volume center [22]. Importantly, most studies do not implicate any change in long-term mortality with postoperative PPM implantation [21,22], although there is some evidence to suggest a survival benefit in early follow-up likely related to prevention of fatal bradyarrhythmia secondary to high-grade AV block [23].

Symptomatic high-grade AV block is the most common indication for PPM implantation following TV surgery due to the close proximity of the AV node at the apex of the PoK to the TV annulus [24]. A clinical observation period of 3–7 days is usually recommended prior to PPM implantation [25]. Complete AV block occurring within 24 h postoperatively and persisting for greater than 48 h is less likely to resolve; early PPM implantation may be pursued in these patients [6,25]. The introduction of new transvalvular RV leads following TV surgery remains controversial. One study of 791 patients undergoing TV repair found that in those who underwent new transvalvular RV leads following TV repair (22%), the presence of a transvalvular lead was an independent risk factor for recurrent TR and late mortality on follow-up [26]. In patients who have undergone TV replacement, repair or TV ring, the hemodynamics immediately post-valve surgery may create limitations for placement of transvenous RV lead due to the acute changes in the regurgitant volume. Another study examining 58 patients undergoing PPM implantation following bioprosthetic TV replacement found no increased risk of significant prosthetic TR [27]. The 2021 ESC Guidelines on cardiac pacing recommend alternative pacing strategies following surgical biological tricuspid valve replacement (TVR) or tricuspid valve ring repair [25]. In patients requiring pacing at the time of TVR, implantation of an epicardial lead is recommended (Class IIa, Level C). For those requiring pacing after TVR, transvenous coronary sinus lead implantation or epicardial ventricular lead placement should be considered and preferred over a transvalvular transvenous approach (Class IIa, Level C) [25].

Accordingly, new transvalvular lead implantation after TV surgery should generally be avoided due to the risk of lead fracture and prosthetic valve dysfunction, particularly in mechanical TV prostheses [6]. In such cases, consideration should be given to coronary sinus leads, epicardial ventricular lead placement, or leadless pacemakers (LPM), which avoid mechanical interference with valve prostheses and have been shown by several smaller studies to be safe and feasible in treating postoperative conduction disorders [28,29,30].

Prophylactic epicardial lead placement at the time of TVR has also been shown to be practical and safe. In a single-center cohort of 80 patients undergoing surgical TVR, 46% required postoperative pacing for AV conduction disturbances. The prophylactic epicardial strategy was associated with an acceptable safety profile, with life-threatening complications occurring in 2.5% of patients [31].

Alternative strategies, such as coronary sinus lead implantation, have also been described; however, feasibility limitations and lead-related complications (4.3–6.2%) must be considered, particularly in patients at high risk for high-grade AV block [32].

Despite this, robust randomized controlled trial data with long-term outcomes follow-up are still lacking [21].

## 4. Transcatheter Tricuspid Valve Interventions

### 4.1. Devices and Outcomes

The uptake of TTVI into formal society guidelines is evolving. The 2025 European Society of Cardiology (ESC)/European Association for Cardio-Thoracic Surgery (EACTS) guidelines provide a Class IIa recommendation for TTVI in high-risk patients with symptomatic severe TR despite optimal medical therapy [14], while the 2020 American College of Cardiology (ACC)/American Heart Association (AHA) guidelines excluded TTVI modalities from any formal recommendation [15]. This gap may be filled in the coming years as TTVI adoption rates continue to rise [33] and the data supporting their efficacy and safety proliferate.

Recent trials have precipitated rapid popularization of novel minimally invasive TTVIs for treatment of severe TR. Tricuspid transcatheter edge-to-edge repair (T-TEER) currently remains the most implemented TTVI globally due to the superior availability of devices [3]. Following the success of TEER in treating mitral regurgitation (MR), this technique was transitively applied for management of severe TR [34] and prompted the eventual release of the TriClip^TM^ system (Abbott Structural Heart, Santa Clara, CA, USA), which was formally approved for treatment of TR by the European Conformity (CE) Mark in April 2020 and by the US Food and Drug Administration (FDA) in April 2024. This decision was informed by the results of the TRILUMINATE Pivotal trial, which found that T-TEER with medical therapy for treatment of severe TR led to significantly greater reductions in TR severity and improvement in quality of life as determined by the Kansas City Cardiomyopathy Questionnaire (KCCQ) score when compared to medical therapy alone [35]. Subsequent analyses demonstrated the benefits in health status following T-TEER to persist with additional improvement at 1 year, with a number needed to treat of 3.5 when compared to medical therapy alone [36]. Two-year outcomes of the TRILUMINATE trial also demonstrated a relative risk reduction for annualized heart failure hospitalization of 28% in the device group when compared to control (HR 0.72, *p* = 0.02) [37]. The PASCAL system (Edwards Lifesciences, Irvine, CA, USA) is an alternative TEER system originally developed for treatment of MR that has also been demonstrated to successfully reduce severity of TR and improve quality of life [38] and received CE Mark approval for treatment of both MR and TR in August 2022, although this device has yet to be formally approved by the FDA for treatment of TR.

Orthotopic TTVR is a more novel approach to the management of severe TR that has gained recognition following reports of its successful implantation through both trans jugular and transfemoral approaches [39,40,41]. The EVOQUE TV replacement system (Edwards Lifesciences, Irvine, CA, USA) marks the first percutaneous transfemoral TTVR system and has demonstrated the capability to significantly reduce TR grade and severity. It was approved for TV replacement by CE Mark in October 2023 and by the FDA in February 2024. The first TRISCEND study was a single-arm, multicenter prospective study utilizing the EVOQUE system and found TTVR to provide sustained TR reduction while improving clinical, functional, and quality of life outcomes at 1-year follow-up [42]. The TRISCEND II pivotal trial subsequently became the first randomized control trial (RCT) to examine TTVR and medical therapy against medical therapy alone for 400 patients with severe symptomatic TR, finding TTVR to improve outcomes with respect to all-cause mortality, New York Heart Association (NYHA) Class, KCCQ overall summary (KCCQ-OS) score, and 6 min walk distance while also carrying increased risk of severe bleeding and new PPM [5]. More recent preliminary data from the TRISCEND II 2-year outcomes trial, when evaluating the study population by crossover status, found TTVR to significantly reduce the combined endpoint of all-cause mortality or heart failure hospitalization when compared to control (36.0% vs. 57.4%, *p* = 0.004), with the relative difference in 2-year mortality being the most prominent (19.1% vs. 44.9%, *p* < 0.001) [43]. This finding is significant in that it constitutes the first time that clinical outcomes data will demonstrate a mortality benefit of TTVI over medical therapy alone. The Intrepid device (Medtronic, Minneapolis, MN) was designed for transcatheter mitral valve replacement and has also been used successfully for TTVR [44], but long-term outcomes data for this indication remain sparse. Finally, while methods targeting TV annular reduction and heterotopic valve replacement devices have been approved for use primarily in Europe, their adoption remains limited, and thus, data supporting their use is similarly constrained [45].

### 4.2. Incidence of Postprocedural Permanent Pacemaker Implantation

The safety profile of T-TEER devices has been demonstrated to be excellent, with data from the TRILUMINATE trial showing no increased risk of TV surgery or new PPM or implantable cardioverter-defibrillator (ICD) placement in the TEER group at 1-year follow-up [35]. Sparse reports in the literature hint at the possibility of late development of new arrhythmias or device-related endocarditis, although true incidence has not yet been reported [46]. Recent 2-year follow-up data from the TRILUMINATE trial also showed comparable rates of conductional abnormalities requiring new PPM implantation between the device group and control (5.5% vs. 4.2% at 2 years) [37].

In contrast, postprocedural conduction abnormalities are one of the major complications of TTVR, with TRISCEND II finding that the need for new PPM implantation was substantially higher in the TTVR group, affecting 17.4% of patients versus 2.3% in the control group (*p* < 0.001) [5]. When examining only patients without PPM at baseline, a new PPM was required in 27.8% of patients in the TTVR group compared to 3.8% in the control group (*p* < 0.001). Much of this increased risk, when compared to other transcatheter procedures, can be attributed to mechanical trauma to the TV-adjacent conduction anatomy secondary to deployment of the transcatheter valve [47]. Certain patient characteristics are also likely to confer increased perioperative risk of conduction abnormalities, including baseline hyperdynamic right ventricular function and contact of the implanted valve with the membranous septum [48]. Longer-term follow-up will be required to determine whether requirement of new PPM following TTVR portends any increase in long-term mortality or heart failure readmission rates, the latter of which has been demonstrated in patients requiring new PPM implantation following surgical TV repair or replacement [21]. The timing of PPM implantation for conduction abnormalities following TTVR may be delayed, allowing for adequate valve endothelialization prior to transvalvular instrumentation; while there is no definitive threshold per guidelines, prior in vitro studies have suggested development of persistent monolayers of endothelial cells within 6–12 days of surgical bioprosthetic valve implantation [49]. Patients who develop clinically significant conduction abnormalities or AV block intra-operatively may be transiently paced over the guidewire or via coronary sinus leads until definitive pacing strategies may be safely pursued. To adequately assess conduction abnormalities following TTVR, telemetry monitoring may be pursued for 3 days post-procedure [39] followed by at least 2 weeks of ambulatory cardiac monitoring. Eventual PPM implantation should consider use of LPM or more permanent coronary sinus pacing to avoid crossing the implanted TV leaflets and thus minimize risk of leaflet dysfunction [50,51].

## 5. Pre-Existing Transvalvular Right Ventricular Leads

### 5.1. Mechanism

It is well recognized that patients with transvalvular CIED leads have more than a twofold higher prevalence of moderate-to-severe TR compared with those without CIEDs [52]. The reported prevalence of TR following CIED implantation varies widely from 7 to 45%, and 10–38% when classified as a ≥2 grade increase in TR severity [53,54,55]. Consequently, these patients carry an increased risk of mortality and heart failure–related hospitalizations [56]. CIED-associated TR is generally categorized into two mechanisms: lead-related or CIED-induced TR. In lead-related TR, the device’s lead directly impairs TV leaflet motion or coaptation, resulting in regurgitation. In contrast, CIED-associated TR (functional) is not accompanied by any imaging evidence of direct leaflet-transvenous lead interaction, and regurgitation instead arises from multifactorial mechanisms such as tricuspid annular enlargement, RV dilation or dysfunction, right atrial enlargement, high burden of RV pacing (>90%), or pulmonary hypertension [57].

CIED-induced TR may arise from iatrogenic injury to the TV apparatus, mechanical interference with TV leaflet mobility and coaptation, or from pacing-related RV desynchrony. Several procedural and technical factors increase the risk of CIED-induced TR [58]. Certain lead implantation techniques, such as direct crossing of the lead into the RV, rather than prolapsing across the valve, may increase the risk of leaflet perforation or laceration. Furthermore, TV passage angle can also predispose entanglement within the leaflets or chordae, specifically impingement on the septal leaflet, which can later develop into fibrous adhesions between the lead and the TV/subvalvular apparatus. Additionally, lead characteristics, including number, thickness, stiffness, and design, can increase the risk, with tined leads shown to be associated with increased risk of TV entanglement [58].

Understanding the underlying mechanism of TR is essential, as it can help guide management strategies. Given the complex anatomy of the TV and the limitations of 2D imaging, distinguishing CIED-induced from lead-associated TR can be challenging. Comprehensive assessment of the TV involves a combination of both transthoracic echocardiography (TTE) and transesophageal echocardiography (TEE) as the mainstay of diagnosing mechanisms of CIED-induced TR.

### 5.2. Pre-Existing Transvalvular Right Ventricular Leads in Tricuspid Valve Surgery

The optimal management of existing RV leads during TV surgery has been unclear, with varying degrees of certainty on the recommendation for removal and replacement with an epicardial pacing system [59]. More recent discourse has centered around the safety and feasibility of maintaining the native TV and positioning the existing RV lead externally to the prosthesis, which has been shown by several case series with long-term follow-up to be effective and well tolerated [59,60]. The risks of device infection are estimated from 0.9% to 1.6% [61,62] and which would necessitate an open surgical approach for lead removal and replacement [6]. In patients with anatomy favorable for surgical TV repair, lead repositioning and a modified cleft repair with approximation of the septal and posterior/inferior TV leaflets with or without annuloplasty have been shown to be safe and effective [63].

### 5.3. Pre-Existing Transvalvular Right Ventricular Leads in Transcatheter Tricuspid Valve Intervention

With the growing awareness of TV disease and an aging population, the use of TTVI is expected to rise. Given the substantial number of patients with pre-existing transvalvular RV leads and concomitant TR, careful consideration of optimal management strategies is essential [53]. A recent joint expert consensus statement from the European Heart Rhythm Association (EHRA) and the European Association of Percutaneous Cardiovascular Interventions (EAPCI) of the ESC provides guidance on managing existing transvalvular RV leads. Management strategies should be individualized depending on the modality of TTVI, the primary mechanism of the TR, and the extent to which the lead contributes to the overall severity of regurgitation [58].

In T-TEER, if the lead is not involved in the pathogenesis of TR, interaction with the lead during the procedure should be minimal with negligible postoperative risk of device detachment or dysfunction [58]. Conversely, in cases where the lead has a causative role in TR, advanced imaging should be pursued to determine whether the lead is fused to a valve leaflet. If there is lead mobility, T-TEER may continue with implied displacement and fixing of the lead into one of the commissures, although excessive interaction between the TEER catheter and the lead should be avoided to prevent entanglement and lead damage [58]. Furthermore, valve recrossing can be challenging depending on the number and location of the implants and necessitates echocardiographic guiding. Irrespective of the role of an existing RV lead in the pathogenesis of TR, lead-related shadowing may present a challenge in accurate imaging and reduce the operator’s ability to place multiple devices or target multiple jets to obtain optimal TR reduction.

Direct percutaneous annuloplasty replicates the principles of surgical prosthetic annuloplasty by targeting annular dilation in functional TR. The Cardioband system has demonstrated favorable clinical outcomes and durable TR reduction [64]. However, annuloplasty can be technically challenging in the presence of a transvalvular lead, particularly when the lead courses near the postero-septal or antero-septal regions, due to limited visualization and the potential need for lead jailing. This requires careful assessment, as lead jailing may result not only in lead dysfunction but also fixed leaflet impingement and worsening TR. Therefore, lead extraction with transcatheter annuloplasty may be considered.

In patients with existing transvalvular RV leads prior to orthotopic TTVR, optimal management of transvalvular leads remains uncertain, with two main strategies currently proposed.

### 5.4. TTVR and Lead Jailing

**Figure 5:**
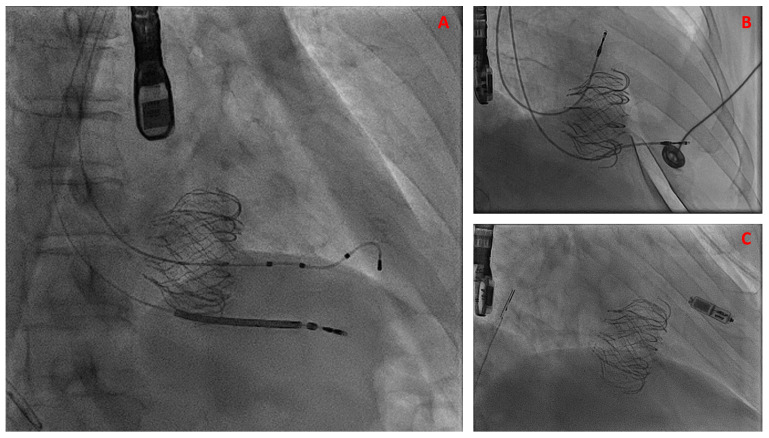
Fluoroscopic Imaging of Cardiac Implantable Electronic Devices with Transcatheter Tricuspid Valve Replacement. Intraprocedural fluoroscopic imaging of (**A**) an implantable cardiac defibrillator coil and (**B**) transvalvular pacemaker lead entrapped against the tricuspid annulus following EVOQUE implantation. (**C**) shows a leadless pacemaker implanted two weeks after transcatheter tricuspid valve replacement for new significant conduction abnormalities.

Lead “jailing” refers to the intentional entrapment of a pre-existing transvalvular lead between the implanted tricuspid prosthesis and the native or surgical annulus during TTVI. One analysis of the Valve-in-Valve Intervention Database found that around 9.4% (n = 31) of patients undergoing TTVR had existing transvenous pacemaker leads transgressing the TV [65]. Fortunately, TTVR in this patient population did not lead to a significant difference in cumulative incidences of death, TV reintervention, and TV dysfunction. Most (n = 28) were performed by jailing the lead with an observed lead failure of 11% [65]. More recently, Mekary et al. reported their real-world experience with entrapped RV leads following TTVR [66]. Among 14 patients with jailed RV leads, a 21% lead failure rate was observed, with 14% mortality at a median follow-up of 10.5 months. Reported causes of death included local CIED infection progressing to endocarditis and sudden cardiac death in a pacemaker-dependent patient. It is worth noting that early data of EVOQUE implantation found no instances of PPM dysfunction with entrapment of existing transvalvular leads [39], although long-term follow-up data is still lacking and lead jailing may preclude lead extraction in cases of infection or delayed complications [67]. A recent study of 52 patients with pre-existing transvalvular leads undergoing TTVR with EVOQUE across 3 medical centers found procedure-related lead complications to be relatively rare, occurring in 4/52 (7.7%) of cases [68]. Complications included immediate lead dislodgement, RV defibrillator lead fracture, and one case of delayed entrapped lead endocarditis, which resulted in patient death partially due to inability to extract the entrapped lead [68]. Additional considerations may be made for existing trans-TV ICD leads, including location of lead entrapment in relation to the shocking coils and whether the extant ICD was indicated for primary or secondary prevention, as patients with known potentially fatal ventricular arrhythmias who are reliant on shock therapy will be at higher risk of adverse events in case of TTVR-related lead malfunction. More research is needed to quantify the extent of long-term risk of lead malfunction in this population. Example images of entrapped RV lead for all TTVI modalities may be found in Figure 5.

### 5.5. TTVR and Transvenous Lead Extraction

Transvenous lead extraction (TLE) may represent an alternate preoperative management strategy for existing trans-TV leads. Early TLE methodology involved simple manual traction that often proved ineffective and risky, carrying high rates of mortality secondary to myocardial avulsion and tamponade [69]. Advances in technique in interval years, however, have led to improved success with studies demonstrating the safety and efficacy of TLE, including old leads with a dwell time of >10 years [7,69,70]. Implementation of newer technologies, including the use of excimer laser sheaths, has also seen increasing uptake and success [7,71]. The European Heart Rhythm Association surveys and the ELECTRa (European Lead Extraction Controlled) Registry (N = 3510) demonstrated that TLE rarely leads to major complications (1.7%) and death (0.5%) [72]. However, when major complications do occur, they may still carry a high risk of mortality. Analysis of the Implantable Cardioverter-Defibrillator Registry reported major complications in approximately 2.3% of cases, of whom 16% required urgent cardiac surgery, with an in-hospital mortality rate of 34% [73]. Notable risk factors for adverse events associated with TLE include female sex, coagulopathy, number of leads to be extracted, old leads with a dwell time of >10 years, and limited operator or center experience [58,74,75]. As a result, TLE may not be readily available at many institutions that lack the staff and procedural subspecialists to safely perform the procedure. Several risk stratification tools have been proposed to aid procedural planning; however, none have been adopted in routine clinical practice [76,77].

Furthermore, TLE may cause direct damage to the valvular/subvalvular tricuspid apparatus, which should be considered when planning TLE before TTVI. Worsening TR (defined as ≥1 grade increase) has been described with TLE as high as 11.5–15%, especially in leads >7 years old [78,79,80]. Iatrogenic leaflet injuries, which usually result in a new flail leaflet, may occur irrespective of the extraction tool used. Identified risk factors for iatrogenic injuries include prolonged lead dwell time and the presence of multiple leads traversing the TV, with procedural complexity often reflecting dense adhesions [58]. Such traumatic injury may compromise the feasibility or effectiveness of subsequent TTVI. Accordingly, comprehensive reassessment after TLE is essential to determine candidacy for TTVI and to select the most appropriate strategy.

Data on the use of TLE alone as a treatment for CIED-induced TR remains limited. Careful assessment of the etiology behind TR is essential, as observational studies suggest that lead removal may reduce TR severity in selected patients. The public comment draft of the 2026 HRS Expert Consensus Statement Update on CIED Lead Management and Extraction assigns a Class IIa recommendation in support of lead removal in selected patients with severe TR when the mechanism is secondary to the existing transvenous lead. This is based on the variable clinical outcomes following TLE, which likely reflects the heterogeneity in TR mechanisms. In a large cohort of 2678 patients, Polewczyk et al. [81] reported a 35% reduction in TR severity among those with lead-related TR, underscoring the importance of accurately evaluating TR etiology. On the other hand, Nazmul et al. [82] demonstrated that significant annular dilation was associated with persistent TR despite extraction, highlighting the need for careful patient selection. Therefore, a multidisciplinary evaluation is essential to assess the risks of TLE, as well as to define the underlying TR mechanism and the anatomic relationship between the lead and the TV [83].

### 5.6. Valve-Sparing Pacing and ICD Alternative Strategies

Valve-sparing pacing strategies have been proposed to minimize the rate of lead-related TR. Long-term ventricular pacing options include coronary sinus pacing, surgical epicardial lead placement, LPM implantation [58]. Coronary sinus pacing may be an appropriate option. However, it is limited by lead instability, phrenic nerve capture, and highly variable capture thresholds [84]. Epicardial pacing avoids endocardial valve interaction but requires surgical pericardial access, which may be difficult in patients indicated for TTVI. In addition, there have been documented higher lead failure rates and often less favorable pacing parameters compared with conventional transvenous systems [58].

LPM systems have shown low procedural and post-implant complication rates and can be implanted after TTVR [51]. However, it does not fully exclude the occurrence of TR, even in the absence of direct leaflet interaction. This is likely related to mechanical interference with the subvalvular apparatus or to pacing mode itself, as seen in a small observational study including 53 patients followed up to 12 months [85]. To minimize valve interaction, devices should be positioned away from the septal annular region [86].

In patients requiring resynchronization therapy, fully leadless cardiac resynchronization therapy (CRT) such as the WiSE-CRTTM (EBR Systems, Sunnyvale, CA, USA) system delivers left ventricular endocardial pacing. His bundle pacing provides more physiologic activation and generally preserves TV function, though direct valve interaction cannot be excluded. LBB area pacing is an alternative for patients with high pacing burden or those with reduced LVEF requiring resynchronization, but careful implantation and valve assessment are required [87]. In addition, there are advances in leadless pacing, including clinical trials with an Abbott Medical left bundle area leadless pacemaker in the high septum that is awaiting clinical approval.

For patients requiring ICD therapy, subcutaneous ICD (S-ICD) or extravascular ICD (EV-ICD) systems represent attractive alternatives that avoid transvalvular leads. Additional alternatives include defibrillation coil placement in the coronary sinus or azygos vein with coronary sinus-based sensing and pacing [86].

Valve-sparing pacing and ICD strategies should be considered in CIED candidates with clinically significant TR who may require future TTVI. A multidisciplinary heart team discussion is essential to select the optimal device strategy while avoiding transvalvular lead placement.

## 6. Conclusions

TV surgery and TTVI have increased due to the growing recognition of the significant morbidity and mortality associated with TR. As these therapies expand, conduction abnormalities remain a clinically significant complication across both modalities, reflecting the intimate 3D relationship between the tricuspid apparatus and the AV conduction axis. In patients requiring pacing therapy, valve-sparing strategies, including LPM and alternative defibrillator systems, should be considered to minimize interaction with the tricuspid apparatus. The optimal management of pre-existing transvalvular leads remains an area of active evolution, with the balance between lead jailing and transvenous lead extraction requiring individualized, multidisciplinary assessment. Future studies are needed to better quantify the long-term risks associated with lead jailing, extraction, and alternative pacing approaches. Therefore, electrophysiologists play an increasingly important role within the structural heart team, contributing to both the prediction and management of post-procedural conduction abnormalities.

## Data Availability

This study is based on previously published data. No new datasets were generated or analyzed.

## Abbreviations

The following abbreviations are used in this manuscript:

ACC	American College of Cardiology.
AHA	American Heart Association.
AV	Atrioventricular.
CE	European Conformity.
CIEDs	Cardiac implantable electronic devices.
CRT	Cardiac resynchronization therapy.
EACTS	European Association for Cardio-Thoracic Surgery.
EAPCI	European Association of Percutaneous Cardiovascular Interventions.
EHRA	European Heart Rhythm Association.
ESC	European Society of Cardiology.
EV-ICD	Extravascular implantable cardioverter-defibrillator.
FDA	Food and Drug Administration.
HiP-CT	Hierarchical phase-contrast tomography.
ICD	Implantable cardioverter-defibrillator.
KCCQ	Kansas City Cardiomyopathy Questionnaire.
LBB	Left bundle branch.
LPM	Leadless pacemakers.
MR	Mitral regurgitation.
NYHA	New York Heart Association.
PoK	Pyramid of Koch.
PPM	Permanent pacemaker.
RCT	Randomized controlled trial.
RBB	Right bundle branch.
RV	Right ventricle.
S-ICD	Subcutaneous implantable cardioverter-defibrillator.
TEE	Transesophageal echocardiography.
TR	Tricuspid regurgitation.
TTE	Transthoracic echocardiography.
TV	Tricuspid valve.
TTVI	Transcatheter tricuspid valve interventions.
TVR	Tricuspid valve replacement.
TLE	Transvenous lead extraction.
T-TEER	Transcatheter edge-to-edge repair.

## References

[1] de Winter R.J. (2022). Tricuspid regurgitation: Remembering ‘the forgotten valve’. Neth. Heart J..

[2] Dreyfus J., Flagiello M., Bazire B., Eggenspieler F., Viau F., Riant E., Mbaki Y., Bohbot Y., Eyharts D., Senage T. (2020). Isolated tricuspid valve surgery: Impact of aetiology and clinical presentation on outcomes. Eur. Heart J..

[3] Hahn R.T. (2023). Tricuspid Regurgitation. N. Engl. J. Med..

[4] Wang N., Fulcher J., Abeysuriya N., McGrady M., Wilcox I., Celermajer D., Lal S. (2019). Tricuspid regurgitation is associated with increased mortality independent of pulmonary pressures and right heart failure: A systematic review and meta-analysis. Eur. Heart J..

[5] Hahn R.T., Makkar R., Thourani V.H., Makar M., Sharma R.P., Haeffele C., Davidson C.J., Narang A., O’neill B., Lee J. (2025). Transcatheter Valve Replacement in Severe Tricuspid Regurgitation. N. Engl. J. Med..

[6] Martins R.P., Galand V., Leclercq C., Daubert J.C. (2018). Cardiac electronic implantable devices after tricuspid valve surgery. Heart Rhythm..

[7] Hahn R.T., Wilkoff B.L., Kodali S., Birgersdotter-Green U.M., Ailawadi G., Addetia K., Andreas M., Auricchio A., Ehlert F., George I. (2024). Managing Implanted Cardiac Electronic Devices in Patients with Severe Tricuspid Regurgitation: JACC State-of-the-Art Review. J. Am. Coll. Cardiol..

[8] Hahn R.T., Weckbach L.T., Noack T., Hamid N., Kitamura M., Bae R., Lurz P., Kodali S.K., Sorajja P., Hausleiter J. (2021). Proposal for a Standard Echocardiographic Tricuspid Valve Nomenclature. JACC Cardiovasc. Imaging.

[9] Tawara S. (1906). Das Reizleitungssystem des Säugetierherzens: Eine Anatomisch-Histologische Studie Über das Atrioventrikularbündel und die Purkinjeschen Fäden.

[10] Miyazaki H., Kibos A., Knight B., Essebag V., Fishberger S., Slevin M., Țintoiu I. (2014). Anatomy and Physiology of the Atrioventricular Node: What Do We Know Today?. Cardiac Arrhythmias.

[11] Tretter J.T., Koneru J.N., Spicer D.E., Ellenbogen K.A., Anderson R.H., Ben-Haim S. (2024). A new dimension in cardiac imaging: Three-dimensional exploration of the atrioventricular conduction axis with hierarchical phase-contrast tomography. Heart Rhythm..

[12] Tretter J.T., Anderson R.H., Ellenbogen K.A., Ben-Haim S. (2025). 3D Anatomy of the Atrioventricular Conduction Axis Reconstructed Relative to Gross Anatomical Landmarks Using Hierarchical Phase-contrast Tomography. Arrhythm. Electrophysiol. Rev..

[13] Padala S.K., Cabrera J.A., Ellenbogen K.A. (2021). Anatomy of the cardiac conduction system. Pacing Clin. Electrophysiol..

[14] Praz F., Borger M.A., Lanz J., Marin-Cuartas M., Abreu A., Adamo M., Marsan N.A., Barili F., Bonaros N., Cosyns B. (2025). 2025 ESC/EACTS Guidelines for the management of valvular heart disease: Developed by the task force for the management of valvular heart disease of the European Society of Cardiology (ESC) and the European Association for Cardio-Thoracic Surgery (EACTS). Eur. Heart J..

[15] Otto C.M., Nishimura R.A., Bonow R.O., Carabello B.A., Erwin J.P., Gentile F., Jneid H., Krieger E.V., Mack M., McLeod C. (2021). 2020 ACC/AHA Guideline for the Management of Patients with Valvular Heart Disease: A Report of the American College of Cardiology/American Heart Association Joint Committee on Clinical Practice Guidelines. J. Am. Coll. Cardiol..

[16] Shinn S.H., Schaff H.V. (2013). Evidence-based surgical management of acquired tricuspid valve disease. Nat. Rev. Cardiol..

[17] Dreyfus J., Juarez-Casso F., Sala A., Carnero-Alcazar M., Eixerés-Esteve A., Bohbot Y., Bazire B., Flagiello M., Riant E., Mbaki Y. (2024). Benefit of isolated surgical valve repair or replacement for functional tricuspid regurgitation and long-term outcomes stratified by the TRI-SCORE. Eur. Heart J..

[18] Songur C.M., Simsek E., Ozen A., Kocabeyoglu S., Donmez T.A. (2014). Long term results comparing mechanical and biological prostheses in the tricuspid valve position: Which valve types are better--mechanical or biological prostheses?. Heart Lung Circ..

[19] Kang Y., Hwang H.Y., Sohn S.H., Choi J.W., Kim K.H., Kim K.B. (2020). Fifteen-Year Outcomes After Bioprosthetic and Mechanical Tricuspid Valve Replacement. Ann. Thorac. Surg..

[20] Patlolla S.H., Saran N., Schaff H.V., Crestanello J., Pochettino A., Stulak J.M., Greason K.L., King K.S., Lee A.T., Daly R.C. (2024). Prosthesis choice for tricuspid valve replacement: Comparison of clinical and echocardiographic outcomes. J. Thorac. Cardiovasc. Surg..

[21] Kassab J., Harb S.C., Desai M.Y., Gillinov A.M., Layoun H., El Dahdah J., El Helou M.C., Nakhla S., Elgharably H., Kapadia S.R. (2024). Incidence, Risk Factors, and Outcomes Associated with Permanent Pacemaker Implantation Following Tricuspid Valve Surgery. J. Am. Heart Assoc..

[22] Ragnarsson S., Taha A., Nielsen S.J., Amabile A., Geirsson A., Krane M., Mörtsell D., Sjögren J., Jeppsson A., Martinsson A. (2023). Pacemaker implantation following tricuspid valve annuloplasty. JTCVS Open.

[23] Jokinen J.J., Turpeinen A.K., Pitkänen O., Hippeläinen M.J., Hartikainen J.E. (2009). Pacemaker therapy after tricuspid valve operations: Implications on mortality, morbidity, and quality of life. Ann. Thorac. Surg..

[24] Herrmann F.E.M., Graf H., Wellmann P., Sadoni S., Hagl C., Juchem G. (2021). Atrioventricular Block after Tricuspid Valve Surgery. Int. Heart J..

[25] Glikson M., Nielsen J.C., Kronborg M.B., Michowitz Y., Auricchio A., Barbash I.M., Barrabés J.A., Boriani G., Braunschweig F., Brignole M. (2021). 2021 ESC Guidelines on cardiac pacing and cardiac resynchronization therapy. Eur. Heart J..

[26] Chang J.D., Manning W.J., Ebrille E., Zimetbaum P.J. (2017). Tricuspid Valve Dysfunction Following Pacemaker or Cardioverter-Defibrillator Implantation. J. Am. Coll. Cardiol..

[27] Eleid M.F., Blauwet L.A., Cha Y.-M., Connolly H.M., Brady P.A., Dearani J.A., Espinosa R.E. (2012). Bioprosthetic tricuspid valve regurgitation associated with pacemaker or defibrillator lead implantation. J. Am. Coll. Cardiol..

[28] Sideris S., Drakopoulou M., Oikonomopoulos G., Gatzoulis K., Stavropoulos G., Limperiadis D., Toutouzas K., Tousoulis D., Kallikazaros I. (2016). Left Ventricular Pacing through Coronary Sinus Is Feasible and Safe for Patients with Prior Tricuspid Valve Intervention. Pacing Clin. Electrophysiol..

[29] Li T.Y.W., Seow S.C., Singh D., Yeo W.T., Kojodjojo P., Lim T.W. (2019). Left ventricular pacing in patients with preexisting tricuspid valve disease. J. Arrhythm..

[30] Vaidya V.R., Dai M., Asirvatham S.J., Rea R.F., Thome T.M., Srivathsan K., Mulpuru S.K., Kusumoto F., Venkatachalam K.L., Ryan J.D. (2019). Real-world experience with leadless cardiac pacing. Pacing Clin. Electrophysiol..

[31] Bazire B., Para M., Raffoul R., Nataf P., Cachier A., Extramiana F., Iung B., Algalarrondo V. (2023). Prophylactic epicardial pacemaker implantation in tricuspid valve replacement. Eur. J. Cardio-Thorac. Surg..

[32] Ahsan S.Y., Saberwal B., Lambiase P.D., Chaubey S., Segal O.R., Gopalamurugan A.B., McCready J., Rogers D.P., Lowe M.D., Chow A.W. (2013). An 8-year single-centre experience of cardiac resynchronisation therapy: Procedural success, early and late complications, and left ventricular lead performance. Europace.

[33] Torres C., Lozier M.R., Davidson C.J., Ailawadi G., Donatelle M., Vedantam K., Kodali S.K., Leon M.B., Beohar N. (2022). In-Hospital Outcomes of Transcatheter Tricuspid Valve Repair: An Analysis From the National Inpatient Sample. J. Soc. Cardiovasc. Angiogr. Interv..

[34] Hao Q., Horton J. (2023). Transcatheter Edge-to-Edge Valve Repair for Tricuspid Regurgitation: CADTH Health Technology Review.

[35] Sorajja P., Whisenant B., Hamid N., Naik H., Makkar R., Tadros P., Price M.J., Singh G., Fam N., Kar S. (2023). Transcatheter Repair for Patients with Tricuspid Regurgitation. N. Engl. J. Med..

[36] Arnold S.V., Goates S., Sorajja P., Adams D.H., von Bardeleben R.S., Kapadia S.R., Cohen D.J. (2024). Health Status After Transcatheter Tricuspid-Valve Repair in Patients with Severe Tricuspid Regurgitation. J. Am. Coll. Cardiol..

[37] Kar S., Makkar R.R., Whisenant B.K., Hamid N., Naik H., Tadros P., Price M.J., Singh G., Schwartz J.G., Kapadia S. (2025). Two-year Outcomes of Transcatheter Edge-to-edge Repair for Severe Tricuspid Regurgitation: The TRILUMINATE Pivotal Randomized Trial. Circulation.

[38] Kodali S.K., Hahn R.T., Davidson C.J., Narang A., Greenbaum A., Gleason P., Kapadia S., Miyasaka R., Zahr F., Chadderdon S. (2023). 1-Year Outcomes of Transcatheter Tricuspid Valve Repair. J. Am. Coll. Cardiol..

[39] Webb J.G., Chuang A., Meier D., von Bardeleben R.S., Kodali S.K., Smith R.L., Hausleiter J., Ong G., Boone R., Ruf T. (2022). Transcatheter Tricuspid Valve Replacement with the EVOQUE System: 1-Year Outcomes of a Multicenter, First-in-Human Experience. JACC Cardiovasc. Interv..

[40] Fam N.P., von Bardeleben R.S., Hensey M., Kodali S.K., Smith R.L., Hausleiter J., Ong G., Boone R., Ruf T., George I. (2021). Transfemoral Transcatheter Tricuspid Valve Replacement with the EVOQUE System: A Multicenter, Observational, First-in-Human Experience. JACC Cardiovasc. Interv..

[41] Fam N.P., Ong G., Deva D.P., Peterson M.D. (2020). Transfemoral Transcatheter Tricuspid Valve Replacement. JACC Cardiovasc. Interv..

[42] Kodali S., Hahn R.T., Makkar R., Makar M., Davidson C.J., Puthumana J.J., Zahr F., Chadderdon S., Fam N., Ong G. (2023). Transfemoral tricuspid valve replacement and one-year outcomes: The TRISCEND study. Eur. Heart J..

[43] Thourani V. Two-Year Outcomes of Transcatheter Tricuspid Valve Replacement for Severe Tricuspid Regurgitation: The TRISCEND II Randomized Trial. Presented at the American College of Cardiology (ACC) Scientific Session.

[44] Blusztein D.I., Hahn R.T., Godoy Rivas C., George I., Kodali S.K. (2023). Transcatheter Tricuspid Valve Replacement with Novel Self-Expanding Valve: Secure Fixation in Insecure Anatomy. JACC Case Rep..

[45] Madhavan M.V., Agarwal V., Hahn R.T. (2024). Transcatheter Therapy for the Tricuspid Valve: A Focused Review of Edge-to-Edge Repair and Orthotopic Valve Replacement. Curr. Cardiol. Rep..

[46] Patrascu A., Binder D., Al Farwan F., Weinmann K., Ott I. (2024). Two ‘firsts’ in a patient with tricuspid valve infective endocarditis following edge-to-edge repair: A case report. Eur. Heart J. Case Rep..

[47] Fischer Q., Ellenbogen K.A., Mittal S., Hausleiter J., Nuche J., Sorajja P., Taramasso M., Thourani V.H., Windecker S., Labbé B. (2025). Atrioventricular Conduction Disturbances in Patients Undergoing Transcatheter Tricuspid Valve Intervention: A Multidisciplinary Consensus. JACC Cardiovasc. Interv..

[48] Le Ruz R., Nazif T., George I., Wan E.Y., Agarwal V., Lebehn M., Rezende C.P., Bartkowiak J., Kim E.K., Tat E. (2025). New-Onset Conductance Disturbances After Transcatheter Tricuspid Valve Replacement: A Mechanistic Assessment. JACC Cardiovasc. Interv..

[49] Fischlein T., Fasol R. (1996). In vitro endothelialization of bioprosthetic heart valves. J. Heart Valve Dis..

[50] Bazire B., Algalarrondo V., Dreyfus J. (2023). Permanent Pacemaker Implantation After Tricuspid Valve Surgery. JACC Case Rep..

[51] Fink T., Eitz T., Sciacca V., Rudolph V., Sohns C., Sommer P., Imnadze G. (2024). Transfemoral leadless pacemaker implantation after interventional or surgical tricuspid valve repair. Europace.

[52] Paniagua D., Aldrich H.R., Lieberman E.H., Lamas G.A., Agatston A.S. (1998). Increased prevalence of significant tricuspid regurgitation in patients with transvenous pacemakers leads. Am. J. Cardiol..

[53] Breeman K.T.N., Peijster A.J.L., De Bruin-Bon H., Pepplinkhuizen S., Van der Stuijt W., De Veld J., Beurskens N., Stuiver M., Wilde A., Tjong F. (2023). Worsening tricuspid regurgitation after ICD implantation is rather due to transvenous lead than natural progression. Int. J. Cardiol..

[54] Trankle C.R., Gertz Z.M., Koneru J.N., Kasirajan V., Nicolato P., Bhardwaj H.L., Ellenbogen K.A., Kalahasty G. (2018). Severe tricuspid regurgitation due to interactions with right ventricular permanent pacemaker or defibrillator leads. Pacing Clin. Electrophysiol..

[55] Vij A., Kavinsky C.J. (2022). The Clinical Impact of Device Lead-Associated Tricuspid Regurgitation: Need for a Multidisciplinary Approach. Circulation.

[56] Höke U., Auger D., Thijssen J., Wolterbeek R., van der Velde E.T., Holman E.R., Schalij M.J., Bax J.J., Delgado V., Marsan N.A. (2014). Significant lead-induced tricuspid regurgitation is associated with poor prognosis at long-term follow-up. Heart.

[57] Saran N., Said S.M., Schaff H.V., Maltais S., Stulak J.M., Greason K.L., Daly R.C., Pochettino A., King K.S., Dearani J.A. (2018). Outcome of tricuspid valve surgery in the presence of permanent pacemaker. J. Thorac. Cardiovasc. Surg..

[58] Deharo J.-C., Dreyfus J., Bongiorni M.-G., Burri H., Defaye P., Glikson M., Lever N., Mangieri A., Mondésert B., Nielsen J.C. (2025). Management of patients with transvalvular right ventricular leads undergoing transcatheter tricuspid valve interventions. A scientific statement of the European Heart Rhythm Association (EHRA) and the European Association of Percutaneous Cardiovascular Interventions (EAPCI) of the ESC endorsed by the Heart Rhythm Society (HRS), the Asian Pacific Heart Rhythm Society (APHRS) and the Canadian Heart Rhythm Society (CHRS). Europace.

[59] Molina J.E., Roberts C.L., Benditt D.G. (2010). Long-term follow-up of permanent transvenous pacing systems preserved during tricuspid valve replacement. Ann. Thorac. Surg..

[60] Lin G., Nishimura R.A., Connolly H.M., Dearani J.A., Sundt T.M., Hayes D.L. (2005). Severe symptomatic tricuspid valve regurgitation due to permanent pacemaker or implantable cardioverter-defibrillator leads. J. Am. Coll. Cardiol..

[61] Klug D., Lacroix D., Savoye C., Goullard L., Grandmougin D., Hennequin J.L., Kacet S., Lekieffre J. (1997). Systemic infection related to endocarditis on pacemaker leads: Clinical presentation and management. Circulation.

[62] Greenspon A.J., Patel J.D., Lau E., Ochoa J.A., Frisch D.R., Ho R.T., Pavri B.B., Kurtz S.M. (2011). 16-year trends in the infection burden for pacemakers and implantable cardioverter-defibrillators in the United States 1993 to 2008. J. Am. Coll. Cardiol..

[63] Raman J., Sugeng L., Lai D.T., Jeevanandam V. (2016). Complex Tricuspid Valve Repair in Patients with Pacer Defibrillator-Related Tricuspid Regurgitation. Ann. Thorac. Surg..

[64] Nickenig G., Weber M., Schüler R., Hausleiter J., Nabauer M., von Bardeleben R.S., Sotiriou E., Schäfer U., Deuschl F., Alessandrini H. (2021). Tricuspid valve repair with the Cardioband system: Two-year outcomes of the multicentre, prospective TRI-REPAIR study. EuroIntervention.

[65] Anderson J.H., McElhinney D.B., Aboulhosn J., Zhang Y., Ribichini F., Eicken A., Whisenant B., Jones T., Kornowski R., Dvir D. (2020). Management and Outcomes of Transvenous Pacing Leads in Patients Undergoing Transcatheter Tricuspid Valve Replacement. JACC Cardiovasc. Interv..

[66] Mekary W., Ibrahim R., Lloyd M.S., Bhatia N.K., Westerman S.B., Shah A.D., Byku I., Gleason P., Greenbaum A., Babaliaros V. (2026). Pacing considerations in patients undergoing transcatheter tricuspid valve replacement: Insights from a tertiary care center. Heart Rhythm..

[67] Gabriels J.K., Schaller R.D., Koss E., Rutkin B.J., Carrillo R.G., Epstein L.M. (2023). Lead management in patients undergoing percutaneous tricuspid valve replacement or repair: A ‘heart team’ approach. Europace.

[68] Peigh G., Al-Kazaz M., Davidson L.J., Gerçek M., Potratz M., Malaisrie S.C., Finke R., Meng Z., Baldridge A.S., Gao J. (2025). Outcomes of Entrapped Right Ventricular Pacing or Defibrillator Leads Following Transcatheter Tricuspid Valve Replacement. JACC Cardiovasc. Interv..

[69] Maytin M., Epstein L.M. (2011). The challenges of transvenous lead extraction. Heart.

[70] Ząbek A., Boczar K., Ulman M., Holcman K., Kostkiewicz M., Pfitzner R., Dębski M., Musiał R., Lelakowski J., Małecka B. (2023). Mechanical extraction of implantable cardioverter-defibrillator leads with a dwell time of more than 10 years: Insights from a single high-volume centre. Europace.

[71] Rinaldi C.A., Diemberger I., Biffi M., Gao Y.-R., Sizto E., Jin N., Epstein L.M., Defaye P. (2023). Safety and success of transvenous lead extraction using excimer laser sheaths: A meta-analysis of over 1700 patients. Europace.

[72] Zucchelli G., Di Cori A., Segreti L., Laroche C., Blomstrom-Lundqvist C., Kutarski A., Regoli F., Butter C., Defaye P., Pasquié J.L. (2019). Major cardiac and vascular complications after transvenous lead extraction: Acute outcome and predictive factors from the ESC-EHRA ELECTRa (European Lead Extraction ConTRolled) registry. Europace.

[73] Sood N., Martin D.T., Lampert R., Curtis J.P., Parzynski C., Clancy J. (2018). Incidence and Predictors of Perioperative Complications with Transvenous Lead Extractions: Real-World Experience with National Cardiovascular Data Registry. Circ. Arrhythm. Electrophysiol..

[74] Hamid S., Arujuna A., Ginks M., McPhail M., Patel N., Bucknall C., Rinaldi C. (2010). Pacemaker and defibrillator lead extraction: Predictors of mortality during follow-up. Pacing Clin. Electrophysiol..

[75] Sidhu B.S., Gould J., Bunce C., Elliott M., Mehta V., Kennergren C., Butter C., Deharo J.-C., Kutarski A., Maggioni A.P. (2020). The effect of centre volume and procedure location on major complications and mortality from transvenous lead extraction: An ESC EHRA EORP European Lead Extraction ConTRolled ELECTRa registry subanalysis. Europace.

[76] Kancharla K., Acker N.G., Li Z., Samineni S., Cai C., Espinosa R.E., Osborn M., Mulpuru S.K., Asirvatham S.J., Friedman P.A. (2019). Efficacy and Safety of Transvenous Lead Extraction in the Device Laboratory and Operating Room Guided by a Novel Risk Stratification Scheme. JACC Clin. Electrophysiol..

[77] Jacheć W., Polewczyk A., Polewczyk M., Tomasik A., Kutarski A. (2020). Transvenous Lead Extraction SAFeTY Score for Risk Stratification and Proper Patient Selection for Removal Procedures Using Mechanical Tools. J. Clin. Med..

[78] Givon A., Vedernikova N., Luria D., Vatury O., Kuperstein R., Feinberg M.S., Eldar M., Glikson M., Nof E. (2016). Tricuspid Regurgitation following Lead Extraction: Risk Factors and Clinical Course. Isr. Med. Assoc. J..

[79] Park S.-J., Gentry J.L., Varma N., Wazni O., Tarakji K.G., Mehta A., Mick S., Grimm R., Wilkoff B.L. (2018). Transvenous Extraction of Pacemaker and Defibrillator Leads and the Risk of Tricuspid Valve Regurgitation. JACC Clin. Electrophysiol..

[80] Coffey J.O., Sager S.J., Gangireddy S., Levine A., Viles-Gonzalez J.F., Fischer A. (2014). The impact of transvenous lead extraction on tricuspid valve function. Pacing Clin. Electrophysiol..

[81] Polewczyk A., Jacheć W., Nowosielecka D., Tomaszewski A., Brzozowski W., Szczęśniak-Stańczyk D., Duda K., Kutarski A. (2022). Tricuspid Valve Damage Related to Transvenous Lead Extraction. Int. J. Environ. Res. Public Health.

[82] Nazmul M.N., Cha Y.M., Lin G., Asirvatham S.J., Powell B.D. (2013). Percutaneous pacemaker or implantable cardioverter-defibrillator lead removal in an attempt to improve symptomatic tricuspid regurgitation. Europace.

[83] O’Gara P.T., Little S.H., Badhwar V., Bhave N.M., Guibone K., Kapadia S.R., Kumbhani D.J., Lindenfeld J., Schaller R.D. (2026). Operator and Institutional Recommendations and Requirements for Tricuspid Interventions: 2026 ACC/AHA/ASE/HRS/STS Expert Consensus Systems of Care Document: A Joint Report of the American College of Cardiology, the American Heart Association, the American Society of Echocardiography, the Heart Rhythm Society, and the Society of Thoracic Surgeons. J. Am. Coll. Cardiol..

[84] Noheria A., van Zyl M., Scott L.R., Srivathsan K., Madhavan M., Asirvatham S.J., McLeod C.J. (2018). Single-site ventricular pacing via the coronary sinus in patients with tricuspid valve disease. Europace.

[85] Beurskens N.E.G., Tjong F.V.Y., de Bruin-Bon R.H.A., Dasselaar K.J., Kuijt W.J., Wilde A.A., Knops R.E. (2019). Impact of Leadless Pacemaker Therapy on Cardiac and Atrioventricular Valve Function Through 12 Months of Follow-Up. Circ. Arrhythm. Electrophysiol..

[86] Hai J.-J., Mao Y., Zhen Z., Fang J., Wong C.-K., Siu C.-W., Yiu K.-H., Lau C.-P., Tse H.-F. (2021). Close Proximity of Leadless Pacemaker to Tricuspid Annulus Predicts Worse Tricuspid Regurgitation Following Septal Implantation. Circ. Arrhythm. Electrophysiol..

[87] Torres-Quintero L., Molina-Lerma M., Cabrera-Borrego E., García-Orta R., Tercedor-Sánchez L., Álvarez M. (2020). His-Bundle Pacing From the Right Atrium in a Patient with Tetralogy of Fallot and a Prosthetic Tricuspid Valve. JACC Clin. Electrophysiol..

